# Ketogenic metabolic therapy for chronic kidney disease – the pro part

**DOI:** 10.1093/ckj/sfad273

**Published:** 2023-11-07

**Authors:** Thomas Weimbs, Jessianna Saville, Kamyar Kalantar-Zadeh

**Affiliations:** Department of Molecular, Cellular, and Developmental Biology, University of California, Santa Barbara, Santa Barbara, CA, USA; Kidney Nutrition Institute, Titusville, FL, USA; Los Angeles County Harbor–UCLA Medical Center, Torrance, CA, USA

**Keywords:** diabetic kidney disease, ketosis, nutrition, obesity, polycystic kidney disease

## Abstract

Ketogenic metabolic therapy (KMT) is a medical nutrition therapy to address certain health and disease conditions. It is increasingly used for many non-communicable diseases that are rooted in abnormal metabolic health. Since chronic kidney disease (CKD) is commonly caused by overnutrition leading to hyperglycemia, insulin resistance and diabetes mellitus, the carbohydrate restriction inherent in KMT may offer a therapeutic option. Numerous studies have found that various forms of KMT are safe for individuals with CKD and may lead to improvement of renal function. This is in contrast to the current standard pharmacological approach to CKD that only slows the relentless progression towards renal failure. Kidney care providers, including physicians and dietitians, are usually not aware of non-standard dietary interventions, including KMT, and often criticize KMT due to common misconceptions and uncertainty about the underlying science, including the common misconception that KMT must involve high protein or meat consumption. This review article discusses the rationales for using KMT, including plant-dominant KMT, for treatment of CKD, clarifies common misconceptions, summarizes the results of clinical studies and discusses why KMT is emerging as an effective medical nutrition therapy (MNT) to consider for patients with kidney disease. KMT, including its plant-dominant versions, can expand a practitioner's kidney health toolbox and will likely become a first-line therapy for CKD in certain CKD-associated conditions such as obesity, metabolic syndrome and polycystic kidney disease.

## WHAT IS KETOGENIC METABOLIC THERAPY (KMT)?

We use the term ‘ketogenic metabolic therapy’ (KMT) instead of ‘ketogenic diet’ to make it clear that we are discussing a form of medical nutrition therapy (MNT) to address certain health conditions related to chronic kidney disease (CKD), in contrast to ‘diet’ or culinary preferences. KMT can be as effective as pharmacological interventions and is a therapeutic option that should be in the armamentarium of every physician and dietitian.

There are different ways of implementing KMT, but the common feature is the goal of switching the body's metabolism to a state of ketosis. Ketosis is a normal physiological state wherein the body utilizes ketones, derived from fat reserves or dietary fat, as opposed to glucose as the main energy source. Upon fasting or carbohydrate restriction, the body enters ketosis after the depletion of glycogen stores when insulin levels become low enough to permit the release of fatty acids from the adipose tissues. Some of the released fatty acids are converted by the liver to the ‘ketones’ β-hydroxybutyrate (BHB) and acetoacetate. The circulating fatty acids and ketones then become the main energy supply for most cells and tissues instead of blood glucose.

Ketosis induced by fasting is a form of KMT. However, continuous fasting can, naturally, not be a long-term solution for an MNT. This led to the development of ketogenic diets >150 years ago that substantially restrict carbohydrates, e.g. ≈20–50 g/day (as opposed to 100–>300 g/day in most societies). To compensate for the reduced energy supply from carbohydrates, the dietary fat intake is increased in typical ketogenic diets. Some ketogenic diets also increase the intake of protein, but this is not a general feature of KMT. The carbohydrate restriction in ketogenic diets leads to lower blood glucose levels, and consequently low insulin levels, depletion of glycogen stores, release of fatty acids from adipose cells and production of ketones by the liver. In contrast to continuous fasting, ketogenic diets can be administered as KMT for long periods of time without leading to nutrient deficiencies because the person under KMT continues eating without other restrictions. However, different forms of fasting, such as periodic fasting and time-restricted eating, can also be employed over time under different KMT modalities.

Fasting is arguably the oldest form of MNT, practiced since time immemorial. KMT using ketogenic diets is not a new therapy but has been employed since prepharmacological times as routine therapy for diabetes mellitus [1] and childhood epilepsy [2]. Clearly, ketogenic diets are not recent ‘fad’ diets as they are sometimes incorrectly labeled.

Due to the physiological effect of KMT in utilizing the body's fat stores, KMT is an effective therapeutic option for overweight and obesity and therefore can affect many associated comorbidities and noncommunicable diseases (NCDs). In addition to inducing fat weight loss, KMT may also have anti-inflammatory effects that appear to be largely due to the ketone BHB. BHB is not only an energy carrier, but is also a potent signaling molecule that activates its receptor, the G protein-coupled receptor GPR109a, at blood levels that occur during ketosis [3]. BHB also inhibits the NLRP3 inflammasome [4] and epigenetically alters gene expression by modifying histones [5].

The use of KMT for NCDs is a hot topic of research today, including in type 2 diabetes (T2D), cardiovascular and kidney disease, cancer, dementias, numerous neurological disorders and mental illnesses including epilepsy, bipolar and autism spectrum disorder, autoimmune diseases including multiple sclerosis and rheumatoid arthritis and many other conditions. We will focus here on the use of KMT for CKD.

## COMMON MISCONCEPTIONS ABOUT KMT

Core training for medical professionals, from dietitians to physicians, on KMT is often limited to the treatment of epilepsy. In nephrology, many clinicians may lack training and education in KMT altogether. Consequently, there are common misunderstandings and biases regarding KMT by dietitians and nephrologists. Many practitioners shy away from utilizing or recommending KMT, as they feel uncertain about the science, the risks and how to avoid high protein diets with high meat intake. Some professionals avoid KMT, considering it unfeasible for their patients with CKD to adopt. Others avoid KMT due to the lack of nutrition professionals trained to assist their patients with MNT. Nephrology professionals also tend to approach ketogenic diets with caution due to the perception of them either being high in protein and driving kidney decline or exacerbating cardiovascular risk due to high red meat and animal fat intake. These assumptions may be incorrect. Below we address some common misconceptions.

### Ketosis versus ketoacidosis

Medical professionals commonly confuse ‘ketosis’ with ‘ketoacidosis’. However, besides the fact that they rhyme, they have nothing in common. Ketosis is a normal physiological metabolic state, characterized by low blood glucose levels and moderate blood ketone levels. In contrast, ketoacidosis occurs primarily in type 1 diabetes mellitus, is characterized by extremely high blood levels of both glucose and ketones and is a serious pathologic condition. MNT monitored nutritional ketosis, induced by fasting or ketogenic diets, does not lead to ketoacidosis.

### Carbs are essential

A common misconception is the incorrect belief that carbohydrates, also commonly abbreviated as carbs, are essential nutrients. As known in biochemistry, they are not. The human body can synthesize all needed carbohydrates. Humans are able to adapt to low carbohydrate intake. It is often mistakenly believed that the body has to ‘run on glucose’ or that the brain requires high blood glucose levels to function. If this were correct, taxonomically higher animals would have long gone extinct because they have evolved the need to go in and out of ketosis due to variable food availability. It is important to remember basic physiology and that the human body stores fat (triglycerides in adipose tissue, liver and other organs) besides glucose (glycogen). The brain is well adapted to utilize ketones over glucose as its energy source [6].

### High protein and high animal foods

Another common misconception is that ketogenic diets must be high protein diets or even animal-based diets. This is incorrect. The only requirement is that carbohydrate intake is restricted sufficiently and that triglyceride intake is correspondingly increased. There certainly are ketogenic diets that may be high in protein or animal-based foods. However, plant-dominant (PLADO) and plant-focused ketogenic diets have been used increasingly in recent years, and KMT can be well consistent with the use of PLADO low protein diets [7] and plant-focused nutrition in diabetic kidney disease (PLAFOND) [8] diet regimens. For example, some of the authors (T.W. and J.S.) have contributed to the creation of a plant-focused ketogenic diet program for the treatment of polycystic kidney disease known as ‘Ren.Nu’ [9].

### Keto diet is the same as a high fat Western diet

Some investigators think that ‘high fat Western’ diets are the same as ketogenic diets. High fat Western diets are frequently used in animal experimentation, where they wreak havoc on the metabolism of rodents [10], but these diets invariably also have a high carbohydrate content that prevents the state of ketosis. By definition, a ketogenic diet must have a very low carbohydrate content and must induce ketogenesis. An illustrative example is a recent press release by the American College of Cardiology reporting on a conference abstract about a study of ‘keto-like’ diets [11]. The diets they investigated (25% of energy from carbohydrates) were not ketogenic. Nevertheless, this and similar uninformed press releases are often reflected in the lay press and touted as proof that ketogenic diets are harmful.

### Investigator/author biases

The field of nutrition research is confounded by investigator biases. This is perhaps because investigators themselves consume different types of foods and may wish to prove that their own dietary preferences are the optimal diet for the medical conditions of others, as seen in notorious opinion fights between vegans and carnivores. Investigators sometimes also have personal religious or ethical tendencies regarding food choices. For example, criticism of ketogenic diets—wrongfully perceived as animal-based diets—often comes from organizations devoted to the prevention of animal cruelty and the promotion of plant-based diets, which is understandable but not scientifically sound. Another significant source of investigator bias can be financial conflicts of interest, e.g. when research studies are funded by certain food industries. Biased investigators continue to publish in the scientific literature, which can create confusion for patients and clinicians who may not have time to extensively research each topic, relying on reviews to guide their evidence-based practice.

In this review, we will not focus on discussing whether animal or plant foods are preferable for CKD, although there is emerging data on PLADO and PLAFOND diets [7, 8]. We will also not opine on any other food or culinary aspects or ethical, religious or environmental sustainability questions, because these concerns may be devoid of scientific discussion on potential benefits of ketosis in CKD.

## WHAT ARE THE RATIONALES FOR CONSIDERING KMT FOR TREATMENT OF CKD?

**Figure 1:**
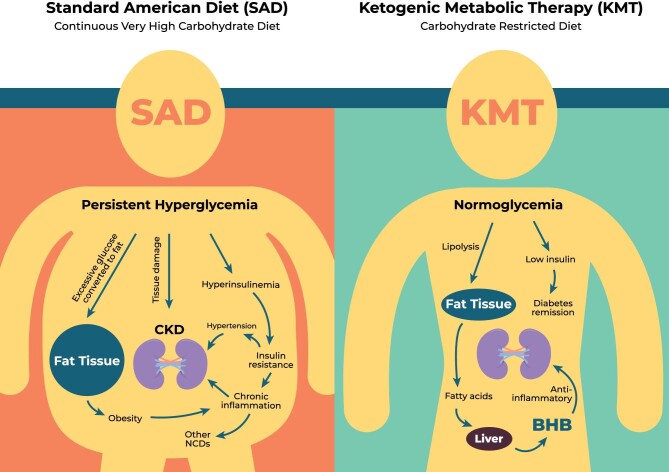
Long-term, persistent hyperglycemia due to the standard American diet (SAD) high in sugar, carbohydrates, calories and highly processed foods leads to chronic hyperinsulinemia, insulin-resistance, hypertension, chronic inflammation, and renal tissue damage. In the presence of insulin, excess carbohydrates are converted to triglycerides and stored in adipose tissues leading to obesity. All of these factors lead to type-2 diabetes and diabetic nephropathy. In contrast, ketogenic metabolic therapy (KMT) reverses these underlying factors. The restriction of carbohydrate intake in KMT leads to normoglycemia and prevents glucose spikes, lowers insulin levels and reverses insulin-resistance and hypertension. The lowering of insulin permits lipolysis by the adipose tissues to release fatty acids which are partially converted to the ketone BHB by the liver that releases BHB into circulation. BHB is the preferred energy substrate of most cells and tissues. In addition to serving as an energy source, BHB is also a signaling molecule that impacts multiple cellular pathways including its strong inhibition of the NLRP3 inflammasome leading to its potent anti-inflammatory properties.

A quintessential rationale is the premise that diabetes is by far the leading cause of CKD. Whereas the exact mechanisms may still be opaque, chronic hyperglycemia has direct effects on the kidneys, eventually causing vascular and tubular damage, chronic inflammation and fibrosis (Fig. 1). If hyperglycemia triggers CKD, it is a logical conclusion that hyperglycemia should be ameliorated as much as possible to prevent further renal health deterioration. KMT is an effective method of decreasing baseline blood glucose levels and reducing spikes. Numerous studies suggest that ketogenic diets are more effective in glycemic control, weight loss and decreasing hypertension than low fat diets [12–14]. A randomized trial comparing head-to-head the Dietary Approaches to Stop Hypertension (DASH) diet to a ketogenic diet over a 4-month period in overweight or obese adults with hypertension and prediabetes or T2D found that both diets had benefits but the ketogenic diet resulted in greater improvements in all outcomes, including improvement of hypertension, glycemic control, hemoglobin A1c (HbA1c) and fat weight loss [15].

T2D is usually regarded as an irreversible, progressive disease since pharmacological treatment usually does not lead to remission. However, clinical experience and numerous studies show that KMT has the potential to achieve remission of T2D [16].

It should be noted that sodium–glucose cotransporter 2 (SGLT2) inhibitors, which are now widely prescribed to CKD patients and often hailed as a breakthrough treatment, work on the same principle as KMT, albeit less effectively [17]. SGLT2 inhibition causes renal excretion of up to 80 g of glucose per day, thereby partially relieving the high carbohydrate burden from patients’ diets. Arguably, the same effect could be achieved by ingesting 80 g less sugar/starch per day, which is the equivalent to about two cans of soda. Using a ketogenic diet, sugar/starch consumption can be further reduced. SGLT2 inhibition also leads to elevation of blood ketone levels, which likely contributes to the benefit [18]. Altogether, SGLT2 inhibition could be considered a ‘light’ version of KMT that, unfortunately, is accompanied by drug-induced adverse effects [19].

The second most common cause of CKD is hypertension, and antihypertensive medication is commonly prescribed for CKD. KMT using ketogenic diets has been shown to significantly lower blood pressure, in some reports even better than the DASH diet that was originally designed to lower blood pressure [15].

Chronic inflammation is a common feature of all forms of CKD and a major contributor to fibrosis and renal decline. As mentioned above, BHB has potent anti-inflammatory properties, and ketogenic diets have been shown to reduce low-grade inflammation in obesity and other conditions [20].

Current nephrology guidelines do not contain KMT-based approaches and hence clinicians may be reluctant to use them in practice due to the incorrect assumption that considering KMT is a deviation from guidelines. However, many pharmacological interventions and dietary recommendations in these guidelines are meant to manage symptoms of CKD, but they may not lead to remission, and CKD almost invariably progresses towards renal failure without effective multimodal integrative interventions that would also include dietary regimens. As discussed below, KMT has been shown to lead to halting of CKD progression or, in some reported cases, may even induce remission. It should be the responsibility of all clinicians to look for additional options beyond guidelines based on patient-centric objectives.

## WHAT IS THE SCIENTIFIC RESEARCH AND CLINICAL EXPERIENCE WITH KMT IN CKD?

Whereas animal studies are not always definitive for human interventions, preclinical studies to date have shown that KMT is beneficial in numerous renal conditions [21]. For example, diabetic nephropathy in mouse models of type 1 and type 2 diabetes was completely reversed by 2-months of maintenance on a ketogenic diet [22]. A ketogenic diet attenuated acute and chronic ischemic kidney injury in rats [23]. Work (by T.W.) showed that intermittent fasting, acute fasting, ketogenic diet or supplementation with BHB resulted in cessation of the progression of CKD in mouse, rat and cat models of polycystic kidney disease and even led to reversal of established CKD [24].

Beyond animal studies, there are several human studies reporting on the safety, feasibility and efficacy of KMT in CKD. In general, KMT proved safe and feasible (relatively high adherence) and improved renal function in these studies. Because protein restriction has traditionally been central to most dietary guidelines for CKD management, it is important to note that many of these studies used a higher protein approach along with ketosis and still saw benefits. This leads us to believe that ketosis was the overshadowing beneficial mechanism and gives room to be more flexible in dietary protein recommendations within a KMT approach.

A randomized controlled trial comparing a 2-year intervention with a ketogenic (low carbohydrate, high fat/protein) diet versus a Mediterranean diet versus a conventional low fat diet in 322 overweight or obese participants with mild–moderate CKD (stages 1–3) showed that the dietary interventions led to weight loss and improved renal function [25]. The ketogenic diet generally had the greatest beneficial effect on renal function, especially in participants with CKD stage 3 [estimated glomerular filtration rate (eGFR) improvement by 7.1 points over 2 years] [25].

In a baseline-controlled, real-life observational prospective study, 92 obese participants were subjected to a ketogenic diet for 15 weeks, including 38 subjects with mild CKD (stage 2) [26]. The intervention resulted in significant fat weight loss, improvement of hypertension, a reduction in total cholesterol and triglycerides and other metabolic improvements. Remarkably, in 27.7% of those with CKD, the intervention led to remission of CKD, leading to an eGFR ≥90 [26]. No safety concerns arose during the study.

A baseline-controlled, retrospective analysis of routine clinical data during the span of 7 years in 143 patients with T2D revealed the following. Patients were prescribed a very low carbohydrate ketogenic diet that not only led to significant weight loss, improvement of glycemic control and HbA1c and a T2D remission rate of 48%, but also led to significant improvements in renal function as shown in the form of creatinine/eGFR and urine albumin:creatinine ratio [27].

A similar retrospective cohort study of longitudinal change in kidney function in >2000 overweight and obese patients was conducted over a span of 19 years. More than 75% of the cohort had CKD (stage 1–3). Treatment with a ketogenic low carbohydrate, high fat and relatively high protein diet improved metabolic parameters, while renal function (creatinine/eGFR) was either unchanged or improved [28].

A randomized controlled study of 40 individuals with T2D and mild–moderate CKD (eGFR >30 ml/min/1.73 m^2^) compared a 6-month intervention with a ketogenic, plant-based fasting-mimicking diet (5 consecutive days per month) to a Mediterranean diet [29]. The KMT intervention led to numerous significant beneficial effects, including improved insulin resistance, glycemic control and HbA1c; weight loss; deprescription of diabetes medication; reduction in microalbuminuria and a slower decrease in eGFR based on cystatin C. In contrast, the Mediterranean diet had no significant effects and led to a continuing decline in eGFR [29].

A recent study compared intervention with a ketogenic diet via continuous remote telemedicine care in 262 individuals with T2D and an eGFR <90 ml/min/1.73 m^2^ with 87 matched individuals receiving usual care over 2 years [30]. The eGFR slope was negative (worsened CKD) in the usual care group but was positive (improved CKD) in the ketogenic intervention group, with a significant difference between groups. Interestingly, subgroup analysis showed that renal function improved most in individuals who achieved sustained nutritional ketosis (consistently ≈1 mM BHB), suggesting a dose-dependent relationship with endogenous ketone concentration [30].

Beneficial effects of KMT on CKD may be independent of the etiology of CKD and act on the progression of the disease rather than only the trigger. Autosomal dominant polycystic kidney disease (ADPKD) is a genetically determined form of CKD and was previously thought to be solely driven by the underlying gene mutations. However, it was shown that the presence of T2D in ADPKD patients associates with faster CKD progression than in individuals with ADPKD alone [31]. Similarly, overweight status and obesity were shown to predict faster CKD progression in individuals with ADPKD [32]. Preclinical research by T.W. on mouse, rat and cat models of ADPKD showed that KMT interventions—time-restricted feeding, acute fasting, ketogenic diet—all led to dramatic slowing of CKD progression [24, 33]. Unexpectedly, ketogenic diets even led to disease reversal in a rat model, which was previously thought to be impossible [24]. Even more remarkably, the beneficial effects of dietary KMT could largely be replicated by supplementing animals with BHB in the drinking water, which suggested that the effects may be due to BHB itself [24].

A retrospective case series study by T.W. on 131 human ADPKD patients who had undergone KMT (mostly ketogenic diets) for an average duration of 6 months showed strong beneficial effects on overweight/obesity reduction, improvement of pain and other ADPKD-related symptoms, significant improvement of hypertension and—unexpectedly at the time—significant improvement in self-reported eGFR [34]. In an explorative, prospective study, about two dozen overweight or obese individuals with ADPKD were randomized to weight-loss diets of either daily caloric restriction or intermittent fasting for 1 year [35]. The interventions led to clinically significant weight loss and potentially slower than expected kidney growth. The results suggested a correlation between the degree of weight loss and slower kidney growth [35]. While ketone levels were, unfortunately, not measured in this study, it must be assumed that those subjects who achieved the greatest weight loss would have had the highest ketone levels during the study.

Based on the fast-emerging research, we (J.S. and T.W.) contributed to the development of a KMT intervention program known as Ren.Nu, (derived from renal nutrition) [9], specifically for individuals with mild–moderate ADPKD. The Ren.Nu program is a 3-month, dietitian-directed, MNT-based group program that combines a plant-focused ketogenic lifestyle approach and the reduction of renal stressors and is supported by the novel medical food KetoCitra that contains exogenous BHB in combination with alkaline citrate. Results from a pilot study of about two dozen individuals with ADPKD [9] and ongoing experience with >150 individuals who have completed the program to date suggests that the intervention is feasible and that many participants experience rapid benefits such as improvement in hypertension and kidney pain. Upcoming controlled clinical trials will evaluate long-term outcomes on metabolic and renal health.

A randomized, prospective pilot trial (KETO-ADPKD) was recently completed in which 66 ADPKD patients were randomized to either of two KMT arms (periodic water fasting or ketogenic diet) or a control group for a 3-month intervention [36]. The study showed high feasibility of the KMT, a significant reduction in body fat and a statistically significant improvement in eGFR (based on serum creatinine and cystatin C) in the ketogenic diet group, despite the relatively short duration of the intervention. Furthermore, the ketogenic diet group showed a trend towards a reduction of kidney volume while not reaching statistical significance.

Altogether, the available clinical studies and experience to date suggest that KMT is feasible in patients with CKD and does not harm the kidneys but rather has beneficial effects including CKD remission. Historically, CKD has been regarded as relentlessly progressive and most nephrologists are not used to seeing CKD remission in their patients with standard pharmacological therapies.

## CONSISTENCY OF KMT WITH OTHER DIETARY REGIMENS FOR CKD

A patient-centered PLADO diet of 0.6–0.8 g/kg/day composed of >50% plant-based sources, administered by dietitians trained in nondialysis CKD care in the form of MNT, has been suggested as a promising diet and consistent with a precision nutrition approach [7]. A type of PLADO diet has been proposed as the PLAFOND diet, with a dietary protein intake of 0.6–0.8 g/kg/day comprised of >2/3 plant-based sources with the lowest glycemic index [8]. The PLADO diets are in contrast to the standard-of-care or traditional renal diets that often include <1/3 plant sources and are low in potassium content [37]. PLADO diets, including PLAFOND, are consistent with KMT regimens, including Ren.Nu [9], given that PLADO diets are not vegan and focus on healthy fats and moderate protein within current guidelines. Indeed, recent data suggest the association of higher fat intake, including saturated fat, with lower mortality [38]. Gluten-free diets have shown salutary effects on certain renal disease, including immunoglobulin A nephropathy (IgAN) and focal segmental glomerulosclerosis (FSGS) [39, 40], hence a vegan diet or a nonspecific PLADO diet may not necessarily be useful or can potentially cause harm if it has higher noncomplex carbohydrates or a higher burden of gluten exposure in IgAN or FSGS. Altogether, we believe that KMT can be consistent with different types of PLADO diets for the management of CKD and its different subtypes.

## ARE THERE RISKS AND SAFETY CONCERNS WITH KMT FOR CKD?

As with any medical or nutritional therapy, there are important elements to consider in order to implement the approach safely. As noted above, some of the perceived risks of KMT are due to misconceptions, including the fear of high protein or high meat intake or the confusion with ketoacidosis or with high fat Western diets. Other concerns that are often raised but can be avoided by clinicians with a trained approach under dietitian-monitored MNT, include the following.

### Kidney stones

A recent meta-analysis estimated that ≈8% of individuals on ketogenic diets develop kidney stones during a follow-up of ≈4 years [41]. It is difficult to judge whether this represents an increase compared with the general population, which has a similar risk of developing kidney stones [42]. However, interestingly, ≈50% of the kidney stones in subjects on ketogenic diets were uric acid stones and an additional 28% were mixed uric acid and calcium stones [41], suggesting a potential link to animal protein intake as a known cause of high uric acid burden. In contrast, calcium oxalate stones are by far the most common stones in the general population. Temporary increases in serum uric acid levels have been reported during the transition into ketosis, which is followed by a long-term decrease below baseline [14]. Urine acidification that occurs in ketosis may increase the risk of forming both uric acid and calcium oxalate stones. Consequently, the Ren.Nu dietary program for ADPKD utilizes an alkalinizing, plant-focused ketogenic diet and the medical food KetoCitra to facilitate the normalization of urine pH and urine citrate levels, along with increased water intake, in order to reduce the risk of kidney stones [9].

### Elevation of low-density lipoprotein cholesterol (LDL-C)

Serum lipid levels and their impact on cardiovascular disease (CVD) risk in a state of ketosis is a controversial topic due to the fact that lipid levels and CVD have primarily been studied in a glucose-fed state. Numerous studies (including those cited above) noted a positive impact on important cardiovascular risk factors including high-density lipoprotein cholesterol levels, triglycerides, body mass index, waist circumference and blood pressure. The only lipid marker that is consistently reported to be elevated in some individuals on ketogenic diets is LDL-C. However, most studies have only reported the standard LDL-C test, which does not differentiate between the harmful small-dense LDL particles that are associated with CVD risk and the benign, large, buoyant LDL particles [43]. A recent meta-analysis found that KMT decreases harmful small dense LDL particles while increasing large LDL particles, suggesting a net beneficial effect on CVD risk [43]. A recent 2-year study on almost 200 individuals with T2D and following a ketogenic diet versus 68 controls on usual care showed no increased CVD risk, and in most cases improved lipids, including the LDL particle profiles [44]. Practitioners should not make recommendations against KMT based solely on effects on LDL-C reported in outdated literature but should focus on monitoring their patients for relevant markers of CVD risk. It should be remembered that dietary interventions do not cause immediate harm, in contrast to some drug-induced toxicities. Therefore, clinicians have the flexibility to monitor their patients over time and make determinations based on the patient's response.

### Keto flu and gastrointestinal problems

‘Keto flu’ is a lay term describing a common occurrence for patients who are entering into ketosis for the first time. It is characterized by a variety of ‘flu-like’ symptoms that may include fatigue, unusual dreams, muscle cramps, headache, brain fog, gastrointestinal symptoms and/or nausea. These symptoms are generally temporary, expected and resolve within a few days. As with any dietary change, gastrointestinal symptoms such as diarrhea or constipation can be due to adaptations of the intestinal microbiome, e.g. in response to changes in fiber or fat intake. Other symptoms such as headaches and cramps are due to electrolyte changes due to depletion of glycogen stores that leads to excretion of associated water. These symptoms can largely be avoided by adequate electrolyte intake, including potassium, magnesium and calcium, as needed during the transition into ketosis.

### Micronutrient deficiency

All dietary regimens contain risks for micronutrient deficiencies with improper composition, heavy use of processed foods and/or a lack of variety. Since ketogenic diets can be implemented with any number of food sources (from plant-based to carnivore and any composition in between), the risk of micronutrient deficiencies largely depends on the composition of the dietary regimen and not on the metabolic state of ketosis. One notable exception may be carnitine, which shuttles activated long-chain fatty acids across the mitochondrial membrane for energy metabolism via β-oxidation. Because of the high demand of using fatty acids for energy, individuals on ketogenic diets may have a higher requirement for carnitine than a vegan/vegetarian diet can provide. This can be monitored with a serum level and can be inexpensively supplemented.

## CONCLUSION

Utilizing KMT for individuals with kidney disease has clinical utility. Multiple studies have shown safety and benefits for patients with CKD, even with higher protein intake. In contrast to the current purely pharmacological dogma for CKD, such as relying on angiotensin-converting enzyme inhibitors, angiotensin receptor blockers and SGLT2 inhibitors, which often leads to almost inevitable progression towards renal failure, KMT may have the potential of CKD remission, which should be investigated in more and larger clinical trials. Kidney health providers, including nephrologists and renal dietitians, are generally not trained in dietary interventions of this kind. Antagonism towards KMT among kidney care practitioners is largely based on misconceptions and outdated studies, such as the fear of ketoacidosis, the belief that carbohydrates are essential nutrients or that KMT equals high protein and high meat intake, and the lack of knowledge about interpreting changes in lipid profiles. There can be many benefits to a patient being guided in an appropriate KMT approach under dietitian-monitored MNT, including improvement in kidney function, glucose management and hypertension and weight loss. A great number of patients can achieve sustainable lifestyle changes and improved outcomes with KMT, including PLADO-based KMT regimens. Indeed, KMT should be considered as a therapeutic intervention by all clinicians engaged in precision nutrition and community nutrition planning for prevention and management of CKD. As with any dietary medical intervention, patients require expert guidance—such as from an experienced dietitian in the form of MNT—an individualized approach that takes into account comorbidities and personal circumstances, monitoring of progress and safety and adjustments based on the patient's experience. Continued research and utilization of KMT for CKD management will lead to its widespread adoption. KMT does not have to be combined with medications and can be implemented inexpensively anywhere in the world.

## Data Availability

No new data were generated or analyzed in support of this research.
